# Regulation of Immune Responses by the Activating and Inhibitory Myeloid-Associate Immunoglobuline-Like Receptors (MAIR) (CD300)

**DOI:** 10.4110/in.2009.9.2.41

**Published:** 2009-04-30

**Authors:** Akira Shibuya, Chigusa Nakahashi-Oda, Satoko Tahara-Hanaoka

**Affiliations:** Department of Immunology, Institute of Basic Medical Sciences, Graduate School of Comprehensive Human Sciences, University of Tsukuba, Ibaraki 305-8575, Japan.

**Keywords:** MAIR, CD300, activating receptor, inhibitory receptor

## Abstract

Activating and inhibitory cell surface receptors play important roles in regulation of immune responses. Recent progress has demonstrated that many inhibitory receptors pair with activating, as well as inhibitory, isoforms, both of whose genes are located in small clusters on a chromosome. We and others identified paired activating and inhibitory immunoglobulin-like receptors, designated myeloid-associated immunoglobulin-like receptors (MAIR) (CD300). MAIR is a multigene family consisting of nine genes on a small segment of mouse chromosome 11. MAIR family receptors are preferentially expressed on myeloid cells, including macrophages, dendritic cells, granulocytes, and bone-marrow-derived cultured mast cells, and a subset of B cells and regulate activation of these cells. Thus, MAIR plays an important role in innate immunity mediated by myeloid cells.

## INTRODUCTION

Activating and inhibitory cell surface receptors play important roles in regulation of immune responses (1,2). The immune inhibitory receptors contain immunoreceptor tyrosine-based inhibitory motif (ITIM) in their cytoplasmic domains. The prototype 6-amino acid sequence for ITIM is (I/V/L/S)-x-Y-x-x-(L/V) (x denotes any amino acid), whose tyrosine is phosphorylated upon ligand binding, providing a docking site for the recruitment of Src homology 2 (SH2)-containing cytoplasmic phosphatases (3,4) and shutting down activation signals by dephosphorylation of intracellular substrates at the earliest steps of the activation response.

In contrast, the activating receptors have a short cytoplasmic domain and contain a charged amino acid residue in the transmembrane region, which is involved in association with immunoreceptor tyrosine-based activating motif (ITAM)-bearing adaptor transmembrane proteins, such as Fc RI or DAP12, or with DAP10 adaptor containing PI3 kinase binding motif (5,6). These adapter proteins are tyrosine phosphorylated by the src family protein tyrosine kinases (PTKs) Syk or Zap70 upon ligand binding, resulting in activation of down stream signaling molecules in lymphoid and myeloid cells. Recent progress has demonstrated that many inhibitory receptors pair with activating, as well as inhibitory, isoforms (2,7), both of whose genes are located in small clusters on a chromosome (8).

Myeloid cells, including neutrophils and macrophages, play an essential role for host defenses against infectious microbial pathogens (9). Myeloid cells are activated through a vast array of cell surface receptors, such as Fc receptors for IgG, β2 integrins, complement receptors, chemokines, cytokine receptors and Toll-like receptors, and mediate phagocytosis, degranulation of anti-microbial substances or secretion of inflammatory mediators. However, how activation of myeloid cells is regulated is incompletely understood. We and others identified and characterized paired activating and inhibitory immunoglobulin-like receptors, designated myeloid-associated immunoglobulin-like receptors (MAIR)-I and MAIR-II and MAIR-IV and MAIR-V, whose extracellular domains are highly conserved with each other, respectively. In this review, we describe molecular and functional characteristics of the MAIR.

## IDENTIFICATION OF THE MAIR

**Figure 1:**
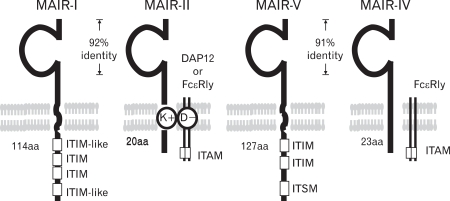
Molecular characteristics of MAIR. MAIR family receptors belong to immunoglobulin superfamily with one immunoglobulin domain in the extracellular portion. MAIR-I and MAIR-II and MAIR-V and MAIR-IV are paired inhibitory and activating receptors, respectively, with high homology in the extracellular portion each other. MAIR-I and MAIR-V contain immunoreceptor tyrosine-based inhibitory motif (ITIM) in their cytoplasmic domains and mediate inhibitory signals. MAIR-II and MAIR-IV associates immunoreceptor tyrosine-based activating motif (ITAM)-bearing adaptor transmembrane proteins Fc RI or DAP12 and mediate activation signals.

To identify novel genes encoding cell surface receptors involved in immune responses by myeloid cells, we performed representative differential analysis (RDA) which is a PCR-based subtractive hybridization, using day 14 fetal livers from PU.1-/-mice lacking myeloid cells and control littermates. Among several cDNA clones unique to myeloid cells identified, we found a novel gene encoding a member of the Ig superfamily, designated *MAIR-I*. The cytoplasmic domain of MIAR-I contains the ITIM-like sequences (VEY^258^STL and LHY^270^SSV, respectively) based on the consensus sequence for ITIMs (I/V/L/SxYxxL/V) (Fig. 1), suggesting that MAIR-I may recruit protein tyrosine phosphatases and mediates inhibitory signals. We also identified a clone encoding the protein, designated MAIR-II, which contains one Ig-like domain with 92% amino acid identity to that of MAIR-I in extracellular domain, a transmembrane region with a charged aa (Lys) and a short (20 aa) cytoplasmic tail (Fig. 1). The MAIR-I and MAIR-II genes are located to the proximal region of E2 band of mouse chromosome 11. MAIR-I and MAIR-II are also named as CLM8/LMIR1 and CLM4/LMIR2/DIgR1, respectively (10-12).

**Figure 2:**
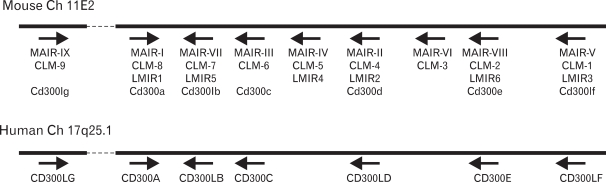
Gene localization of MAIR family. Mouse and human MAIR family genes are mapped on chromosome 11 and 17, respectively.

By screening a database for the mouse genome, we and others found that MAIR-I and MAIR-II are members of a multigene family consisting of nine genes on a small segment of mouse chromosome 11 (11,13) (Fig. 2).

**Figure 3:**
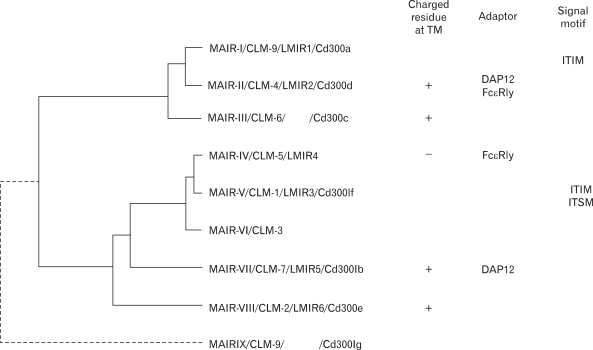
Molecular phylogenetic tree of MAIR gene family. Phylogenetic analysis of the MAIR family genes was performed by using the UPGMA method of GENETYX-MAC software (Software develoment, Tokyo, Japan). MAIR-II, MAIR-III, MAIR-VII and MAIR-VIII contained a charged amino acid in the transmembrane portion, and some of them associate with ITAM-bearing adaptor. MAIR-I and MAIR-V contains ITIM in the cytoplasmic portion.

We cloned all the full-length cDNAs of the MAIR family genes other than MAIR-I and MAIR-II by PCR from the spleen of C57BL/6 mice and designated them as MAIR-III to MAIR-IX, based on a phylogenetic tree analysis (Fig. 3). We found that one of the genes, MAIR-IV, has a short cytoplasmic tail (24 aa) with no signaling motif and a negatively charged glutamic acid (E) in its transmembrane (TM) region (Fig. 1). The Ig-like domain of MAIR-IV in the extracellular portion has 91% identity with that of MAIR-V at the amino acid level. In contrast to MAIR-IV, MAIR-V has a long cytoplasmic tail containing two consensus immunoreceptor tyrosine-based inhibitory motif (ITIM), suggesting that MAIR-IV and MAIR-V constitute paired activating and inhibitory receptors (Fig. 1). MAIR-IV and MAIR-V were also named as CLM5 and CLM1/DIgR2 (11,14,15), respectively. The MAIR family was found to be a murine counterpart of the human CMRF-35 (CD300) family (16-18), which is located on human chromosome 17, syntenic region of mouse chromosome 11 (Fig. 2).

## EXPRESSION AND FUNCTION OF MAIR-I AND MAIR-II, PAIRED INHIBITORY AND ACTIVATING RECEPTORS, RESPECTIVELY

MAIR-I is expressed on the majority of myeloid cells, including macrophages, dendritic cells, granulocytes, and bone-marrow-derived cultured mast cells, and a subset of B cells, but neither on T nor NK cells (19). In contrast, MAIR-II protein is detected only on cell surface of subsets of B cells and peritoneal macrophages (19,20).

MAIR-I contains the ITIM sequences in the cytoplasmic domain and inhibits IgE-mediated degranulation from mast cells (19). Analyses by using the transfectant of rat basophil leukemia RBL-2H3 expressing wild type or variable mutant MAIR-I at Y^233^, Y^258^, Y^270^ and/or Y^299^ demonstrated that both Y^258^ and Y^270^, but not Y^233^ and Y^299^, were phosphorylated and recruits SHP-1and SHIP upon cross-linking of MAIR-I, which were essentially required for inhibition of IgE-mediated degranulation from the RBL-2H3 transfectant (21).

MAIR-II associates with the immunoreceptor tyrosine-based activation motif-bearing adaptor DAP12 and stimulates pro-inflammatory cytokines and chemokine secretions from peritoneal macrophages (19). However, we found that cross-linking MAIR-II with monoclonal antibody induced secretion of significant amount of the inflammatory cytokines TNF-α and IL-6 from DAP12-/- as well as wild type peritoneal macrophages. Further studies demonstrated that MAIR-II associates with not only DAP12 but also FcRγ chain homodimers in peritoneal macrophages (20). These findings present the first case of an activating receptor that uses either DAP12 or FcRγ chain as a signaling adapter.

## EXPRESSION AND FUNCTION OF MAIR-IV AND MAIR-V, PAIRED ACTIVATING AND INHIBITORY RECEPTORS, RESPECTIVELY

MAIR-IV was preferentially expressed on Gr-1^high^/Mac-1^+^ neutrophils from the peripheral blood, bone marrow, peritoneal cavity and spleen. MAIR-IV was also expressed on Mac-1^+^/Gr-1^-^ macrophages from the spleen and bone marrow and CD11c^+^ dendritic cells (DC), but not on T, B, NK cells or bone marrow-derived cultured mast cells (13). MAIR-IV contains a short cytoplasmic tail with no signaling motif, suggesting that MAIR-IV, like MAIR-II, mediates activating signals via ITAM-containing adapter proteins, such as FcRγ chain or DAP12. DAP12 associates with several activating NK receptors and myeloid cell-specific receptors, including human and mouse TREM-1, TREM-2, MDL-1, and PILRβ, SIRP β1, mouse TREM-3 and CD200R3, CD200R4 and mouse MAIR-II, all of which contain a positively charged amino acid, such as lysine (K) or arginine (R), in their TM region (22). In contrast, FcRγ chain is also able to associate with receptors, such as FcγRIIIA and FcεRI, which do not contain a positively charged amino acid in the TM region (23). Because MAIR-IV does not possess a positively charged amino acid in the TM region, FcRγ chain might be a partner of MAIR-IV. In fact, MAIR-IV associate with FcRγ chain in peritoneal macrophages as well as transfectants expressing MAIR-IV (13,14). Because MAIR-IV associates with FcRγ chain, it may mediates an activating signal in neutrophils. Stimulation of MAIR-IV with plate-coated F(ab')_2_ fragments of anti-MAIR-IV mAb induced TNF-α and IL-6 secretion from neutrophils or peritoneal exudatative cells, indicating that MAIR-IV is a novel activating receptor on neutrophils.

MAIR-V (CD300LF) was expressed on macrophages, but not on B cells, T cells or granulocytes, derived from the spleen and peritoneal cavity (24). Cross-linking MAIR-V with anti-MAIR-V monoclonal antibody induced cell death in peritoneal macrophages as well as in several transfectants expressing MAIR-V.

Scanning electron microscopy revealed loss of blebs from the surface of the dead cells mediated by MAIR-V, a morphological feature similar to that observed in apoptotic cells. However, further studies revealed that MAIR-V mediates caspase and ER stress-independent cell death by a novel mechanism.

## CONCLUDING REMARKS

MAIR family receptors are preferentially expressed on myeloid cells, including macrophages, dendritic cells, granulocytes, mast cells, and a subset of B cells and regulate activation of these cells. It is not clear at present how these activating and inhibitory receptors cooperate each other for regulation of immune responses by myeloid cells. To understand the role of MAIR family receptors in immune responses, it is essentially required to identify their ligands and characterize the receptor-ligand interaction *in vivo*.
